# Supplementary material for: COPD Patients Exhibit Distinct Gene Expression, Accelerated Cellular Aging, and Bias to M2 Macrophages
Source: Int J Mol Sci. 2023 Jun 8;24(12):9913. doi: 10.3390/ijms24129913 (PMC10297859; doi:10.3390/ijms24129913)
Supplement: Supplementary file 1 [file ijms-24-09913-s001.zip › ijms-2394406-supplementary.pdf]

Supplementary material

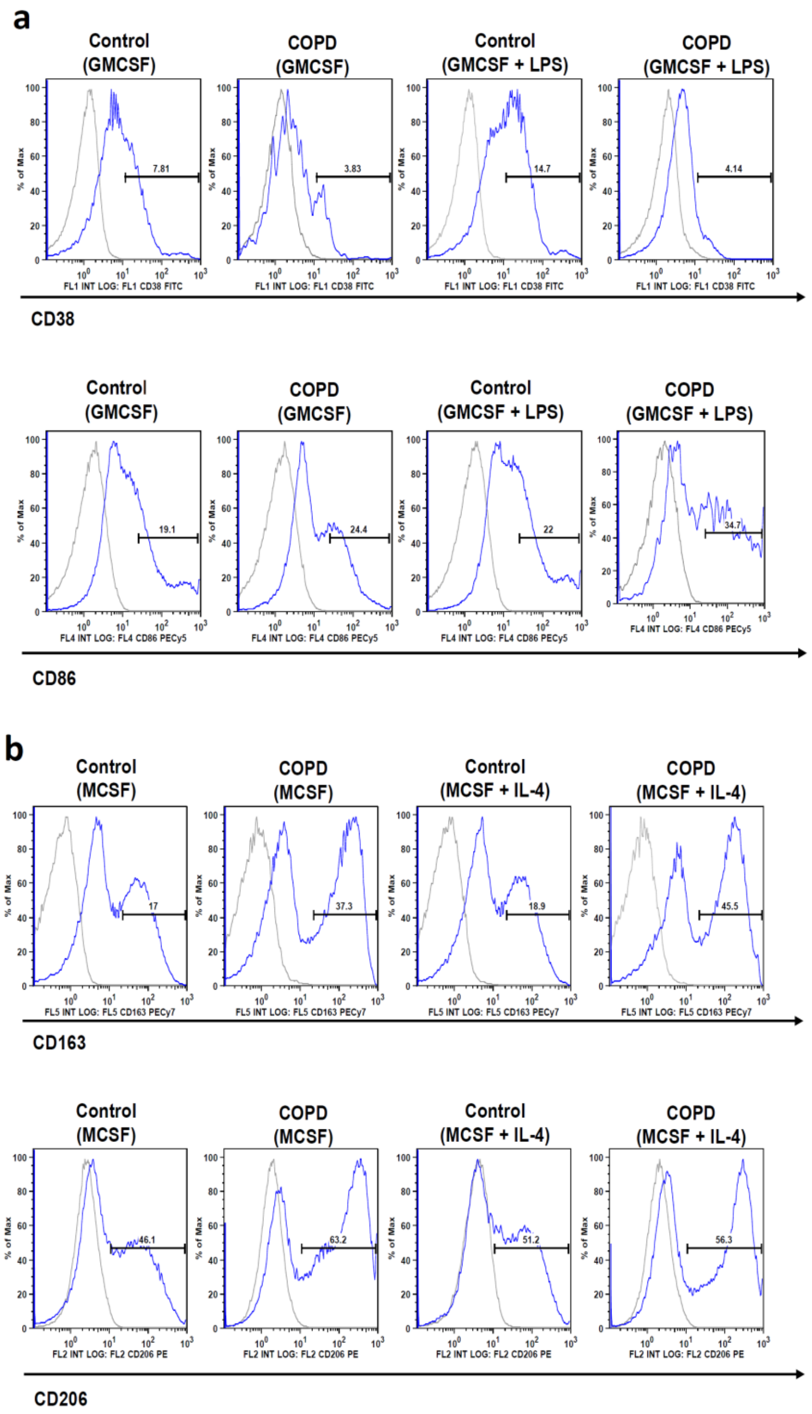

Figure S1. Representative histograms of positive cells for CD38 or CD86 **(a)** and CD163 or CD206 **(b)** in cultured macrophages (with GMCSF or MCSF) derived from peripheral blood monocytes in non-smoking controls (n = 10) and COPD patients (n = 16), stimulated or not with LPS (A) or IL-4 (B), by flow cytometry. Blue lines indicate antibody-labelled cells and gray lines indicate unlabeled cells.

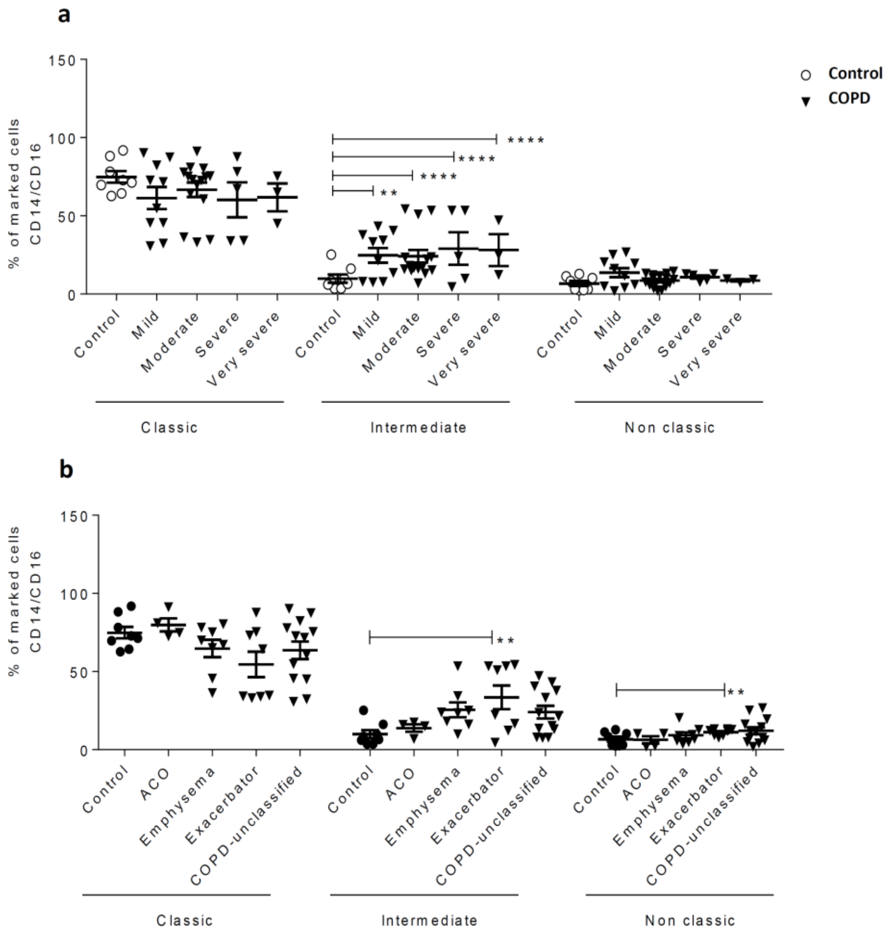

Figure S2. Subtypes of classical monocytes ($CD14^{++}/CD16^{-}$), intermediate monocytes ($CD14^{+}/CD16^{+}$) and non-classical monocytes ($CD14^{+}/CD16^{++}$) were determined by flow cytometry in non-smoking controls and COPD patients with different: **(A)** graveness (mild, n = 10; moderate, n = 15; severe, n = 6 and very severe, n = 4); and **(B)** phenotypes (ACO, n = 4; emphysema, n = 10; exacerbator, n = 8 and COPD- unclassified (n = 13). Statistics were performed using one-way analysis of variance (ANOVA), followed by a Bonferroni test. Black circles represent healthy controls and black triangles represent COPD patients. Data represent mean ± standard error of mean. Significance of p-values: ** p <0.01 e * *** p<0.0001 compared to control.

Materials and Methods

Characterization of the Peripheral Blood Monocytes Profile

Whole peripheral blood (100 μL) was incubated with 5 μL of anti-CD14-FITC, anti-CD16-PE and anti-HLDR-PC5 antibodies (Beckman Coulter, California, USA) for 10 minutes in the dark. Then, 2 mL of red blood cell lysis buffer (BD Biosciences, Franklin Lakes, NJ, USA) were added for 10 minutes; tubes were centrifuged at 1700 *rpm* for 5 min at 20 °C; the supernatant was discarded and the pellet resuspended in PBS1x. After further centrifugation for 5 min, the cell pellet was resuspended in 500 μL of PBS1x with formalin (1%) to fix the cells. The acquisition was performed using a FACS Calibur cytometer (BD Biosciences, Franklin Lakes, NJ, USA), obtaining 200,000 events. Lymphocytes were excluded due to the absence of CD14; NK cells and neutrophils were excluded due to the lack of HLA-DR. Data were analyzed using the Kaluza v5 (Beckman Coulter, California, USA). The results were expressed in individual values (%). Compensation was performed using single-color fluorochrome antibodies for PE and FITC, respectively.
